# New different origins and evolutionary processes of AP2/EREBP transcription factors in *Taxus chinensis*

**DOI:** 10.1186/s12870-019-2044-z

**Published:** 2019-10-07

**Authors:** Meng Zhang, Ying Chen, Xiaofei Jin, Yuxin Cai, Yuanyuan Yuan, Chunhua Fu, Longjiang Yu

**Affiliations:** 1 Department of Biotechnology, Institute of Resource Biology and Biotechnology, College of Life Science and Technology, Huazhong University of Science and Technology, No.1037 Luoyu Road, Wuhan, 430074 People’s Republic of China; 2 Key Laboratory of Molecular Biophysics Ministry of Education, College of Life Science and Technology, Huazhong University of Science and Technology, No.1037 Luoyu Road, Wuhan, 430074 People’s Republic of China

**Keywords:** *Taxus chinensis*, APETALA2/ethylene response element binding protein transcription factors, TcA3Bz1, Evolutionary processes

## Abstract

**Background:**

*Taxus* spp. produces the anticancer drug, taxol, and hence is planted as an industrial crop in China. APETALA2/ethylene response element binding proteins (AP2/EREBPs) are the key regulators of plant development, growth, and stress responses. Several homologues control taxol biosynthesis. Identifying the AP2/EREBP proteins from *Taxus* is important to increase breeding and production and clarify their evolutionary processes.

**Results:**

Among the 90 genes from multi *Taxus chinensis* transcriptome datasets, 81 encoded full-length AP2-containing proteins. A domain structure highly similar to that of angiosperm AP2/EREBPs was found in 2 AP2, 2 ANT, 1 RAV, 28 dehydration-responsive element-binding proteins, and 47 ethylene-responsive factors contained, indicating that they have extremely conservative evolution processes. A new subgroup protein, TcA3Bz1, contains three conserved AP2 domains and, a new domain structure of AP2/EREBPs that is different from that of known proteins. The new subtype AP2 proteins were also present in several gymnosperms (*Gingko biloba*) and bryophytes (*Marchantia polymorpha*). However, no homologue was found in *Selaginella moellendorffii*, indicating unknown evolutionary processes accompanying this plant’s evolution. Moreover, the structures of the new subgroup AP2/EREBPs have different conserved domains, such as B3, zf-C3Hc3H, and agent domains, indicating their divergent evolution in bryophytes and gymnosperms. Interestingly, three repeats of AP2 domains have separately evolved from mosses to gymnosperms for most of the new proteins, but the AP2 domain of Gb_11937 has been replicated.

**Conclusion:**

The new subtype AP2/EREBPs have different origins and would enrich our knowledge of the molecular structure, origin, and evolutionary processes of AP2/EREBP transcription factors in plants.

## Background

*Taxus* spp. is an ancient gymnosperm that could produce a secondary metabolite, taxol (generic Paclitaxel), the most used clinical anticancer drugs [1–3]. *Taxus* spp. is currently the main source of taxol and its precursors and thus is widely planted as an industrial crop in many provinces of China. However, the taxol content in *Taxus* spp. is extremely low, and the biosynthesis pathway is highly complex and requires 19–20 enzymatic steps, leading to a taxol supply shortage.

Regulating secondary metabolite biosynthesis is a promising way to guide breeding, genic manipulation, and planting of industrial crops [4]. Our previous work identified two AP2/EREBPs proteins, TcERF12 and TcERF15, that could positively and negatively regulate the expression of *TASY* gene, which encodes the first committed step enzyme in the taxol biosynthesis pathway [5]. Therefore, identifying AP2/EREBP transcription factors in *Taxus* spp. would facilitate the screening of candidate regulators for taxol biosynthesis.

AP2/EREBP transcription factors play various roles in plants. AP2/EREBP superfamily proteins are divided into six subgroups, namely, APETELA2 (AP2), AINTEGUMENTA (ANT), related to ABI3/VP1 (RAV), dehydration-responsive element-binding protein (DREB), ethylene-responsive factor (ERF), and soloist in most plants [6, 7]. AP2 and ANT proteins mainly function as key developmental regulators in reproductive and vegetative organs and lateral organ development [8, 9]. As negative regulators, RAV proteins mediate plant defense during abiotic and biotic stress [10–12]. DREBs usually function in cold-, drought- and heat-stress responses, and ERFs are often linked to biotic stress responses, such as pathogen attack and methyl jasmonate and ethylene application [13, 14]. Thus, the identification of AP2/EREBP transcription factors is meaningful for further cultural practices.

AP2/EREBP is a highly conserved superfamily in angiosperms, but its transcription factors in gymnosperm plants have not been systematically studied. Gymnosperms are more ancient but as important as angiosperms and often provide new understandings on the evolutionary processes of genes [15]. Thus, *Taxus* spp. would be a valuable material to study the evolution processes of AP2/EREBP proteins in gymnosperms.

We integrated multi transcriptome datasets of *T. chinensis* related to taxol biosynthesis for the identification of AP2/EREBP transcription factors [2, 15–17]. More than 100 Gb sequenced data were used to ensure that most AP2/EREBP proteins were obtained by hidden Markov model (HMM) search. Only the genes encoded with at least a complete AP2 domain were employed for further analysis. AP2/EREBP proteins were also obtained from angiosperms (*Arabidopsis thaliana*), gymnosperms (*Picea abies*, *Ginkgo biloba*, *Pinus tadae*, and *Gnetum montanum*), and bryophytes (*Physcomitrella patens* and *Selaginella moellendorffii*) to clarify the evolutionary differences. Finally, the expression patterns of these TcAP2/EREBP proteins were analyzed and clustered with taxol biosynthesis genes. Results showed that the evolution of AP2/EREBP transcription factors is highly divergent among higher plants and provided interesting findings on the evolution of AP2/EREBP proteins.

## Methods

### Plant materials and transcriptome datasets

The *T. chinensis* cells were induced from 2-years-old Chinese yew (*Taxus chinensis* (Pilger) Rehd*.*) at May 2005 and subcultures with 62# medium [18]. A series of transcriptome datasets was sequenced by BGI (Shenzhen, China); some of these data were previously reported, such as NA/CA [16] and MJ treatment [17]. Two unpublished datasets with an individual size of 36 Gb and the re-assembled transcriptome datasetfrom public reports were also used [15, 19].

### Identification of AP2/EREBP proteins in *T. chinensis*

The transcriptome data in the present study originated from the database of our previous report that was expanded by adding several new datasets such as miR5298OE and WRKY47OE (two genes, miR5298 and WRKY47 involve in taxol biosynthesis were over-expressed in *Taxus* cells and their transcriptome data were high-throughput sequenced; unpublished results) [2, 16, 17, 19]. A large dataset is helpful to obtain enumerous full-length genes.

The AP2/EREBP proteins of *T. chinensis* were identified by HMM search in the HMM v3.1b2 (http://hmmer.org/) by hmmsearch in the HMM v3.1b2 (http://pfam.xfam.org/) using the Hidden Markov Model (HMM) of AP2 domain (PF00847) with a cut-off score of 1e-5 [20]. All nucleotide sequences obtained from various transcriptome datasets were further reassembled by CAP3 (http://doua.prabi.fr/software/cap3) [21], and redundancy was removed by CD-HIT-est with sequence identity cut-off of 0.98 (http://weizhong-lab.ucsd.edu/cdhit-web-server/cgi-bin/index.cgi?cmd=cd-hit-est) [22]. The sequences that could not be extended were aligned with the NR database to identify their conserved domains (AP2 domain, B3 domain, and others) by using online Blastx with default parameters (https://blast.ncbi.nlm.nih.gov/Blast.cgi). ORF finder (https://www.ncbi.nlm.nih.gov/orffinder/) was employed to obtain the full-length deduced protein sequences. For the genes without an intact ORF, the aligned sequences that contained AP2 domains were utilized for further bioinformatics analysis. All nucleotide and amino acid sequences could be downloaded as supplementary files.

### Obtaining AP2/EREBP proteins from *A. thaliana*, *P. patens* and S. moellendorffii, *P. abies*, and *G. biloba*

*Arabidopsis* AP2/EREBP proteins were searched from the plnTFdb database (http://plntfdb.bio.uni-potsdam.de/v3.0/) [23]. The protein databases of *P. patens* and *S. moellendorffii* were downloaded from JGI (https://phytozome.jgi.doe.gov/pz/portal.html#). The protein sequences of *P. abies* and *P. tadae* were obtained from ConGenIE (http://congenie.org/), those of *G. biloba* were downloaded from Giga (http://gigadb.org/dataset/100209), and those of *G. montanum* were acquired from DRYAD (https://datadryad.org/resource/doi:10.5061/dryad.0vm37.2). HMM search was used to screen AP2/EREBP proteins that contain an AP2 domain with a cut-off score of 1e-5. The HMM file (PF02362, http://pfam.xfam.org/family/B3) was used to identify RAV proteins containing a B3 domain in addition to the AP2 domain from AP2/EREBP proteins.

### Sequence alignment and phylogenic and motif analysis of conserved AP2 domains

The proteins were aligned with MAFFT v7.312 (http://mafft.cbrc.jp/alignment/software/) [24] and online ClustalW with default parameters and colored by EsPript 3.0 (http://espript.ibcp.fr/ESPript/cgi-bin/ESPript.cgi) [25]. MEGA 5.0 was used to construct a neighbor-joining tree with these sequences of AP2 domain based on the JTT model after 1000 bootstrap resampling [26]. The tree was validated by phyML 3.1 (http://www.atgc-montpellier.fr/phyml) [27]. When the ML and NJ trees were similar, they were used as the phylogenic tree and colored by FigTree V1.4.3 (http://tree.bio.ed.ac.uk/software/figtree/). The specific logo of AP2 domain from various plants was generated using MEME Suite V5.0.2 webtools in the classic motif discovery mode (http://meme-suite.org/tools/meme) [28].

### Phylogenic analysis of full-length ERF transcription factors

ERF transcription factors are AP2/EREBP proteins that contain only an AP2 domain and constitute the biggest subfamily of the AP2/EREBP family. The full-length ERF proteins of *T. chinensis* and other plants were phylogenetic analyzed to clarify the differences of ERFs in *Taxus* and other plants. MAFFT was used for alignment, and MEGA 5.0 was used to construct the tree based on the JTT model with 1000 bootstrap resampling. The tree was validated by phyML 3.1 (http://www.atgc-montpellier.fr/phyml) [27]. When the ML and NJ trees were simialr, they were used as the phylogenic tree and colored by FigTree V1.4.3 (http://tree.bio.ed.ac.uk/software/figtree/). B3 ERFs containing EDLL motif were aligned using ClustalW with protein weight matrix “Identity”. phyML and FigTree were used to validate and color the tree.

### Motif analysis of ERF transcription factors

The full-length amino acid sequences of *T. chinensis* AP2/EREBP transcription factors, which have intact ORFs and other AP2/EREBP proteins, were analyzed by MEME software with default parameters to identify new and specific motifs in various plants. Domain and motif patterns were illustrated by TBtools (http://cj-chen.github.io/tbtools) [29] and WebLogo (http://weblogo.berkeley.edu/logo.cgi).

### Expression patterns of *T. chinensis* AP2/EREBP transcription factors

*Taxus spp*. is famous for its anticancer metabolite, taxol. Two ERF factors, namely, TcERF12 and TcERF15, regulate taxol biosynthesis. Thus, the known taxol biosynthesis genes were added into the heatmap to clarify the potential regulating roles of AP2/EREBP factors on taxol biosynthesis. Five transcriptome data were obtained from the samples with remarkably different taxol contents. For instance, NA comprised newly induced and accumulating *Taxus* cells, whereas CA comprised long-term subculture cells [16]. Gene expression was calculated by FPKM (NA/CA, GA-treated), TPM (miR5298bOE and WRKY47OE); two genes, miR5298 and WRKY47 involve in taxol biosynthesis were over-expressed in *Taxus* cells and their transcriptome data were high-throughput sequenced), and RPKM (MeJA-treated). Values of log2 (Expression ratio) were used to generate and cluster the heatmap using Morpheus with default parameters (https://software.broadinstitute.org/morpheus). Co-expression coefficient values were calculated using the CORREL formula in Excel.

## Results

### Ninety AP2/EREBP genes were identified in *T. chinensis* transcriptome datasets

Although many unigenes were annotated as AP2/EREBP transcription factors according to the Blast results, only the genes that could encode a full-length AP2 domain were considered. After redundancy removal, sequence assembly, and elongation, 93 genes with a length ranging from 206 bp to 6951 bp could encode at least one full-length AP2 domain (Additional files 1 and 4). In recent studies on AP2/EREBP, nearly 90 genes were found using only transcriptome data [30, 31]. A few *Taxus* AP2/EREBPs were not found, indicating that the expression of several AP2/EREBPs is strictly regulated.

### AP2-, ANT-, and RAV-type AP2/EREBP proteins were minorities in *T. chinensis*

Among the 90 genes, 81 unigenes could encode full-length AP2/EREBP proteins ranging from 146 aa to 1955 aa (Additional file 1).

**Fig. 1:**
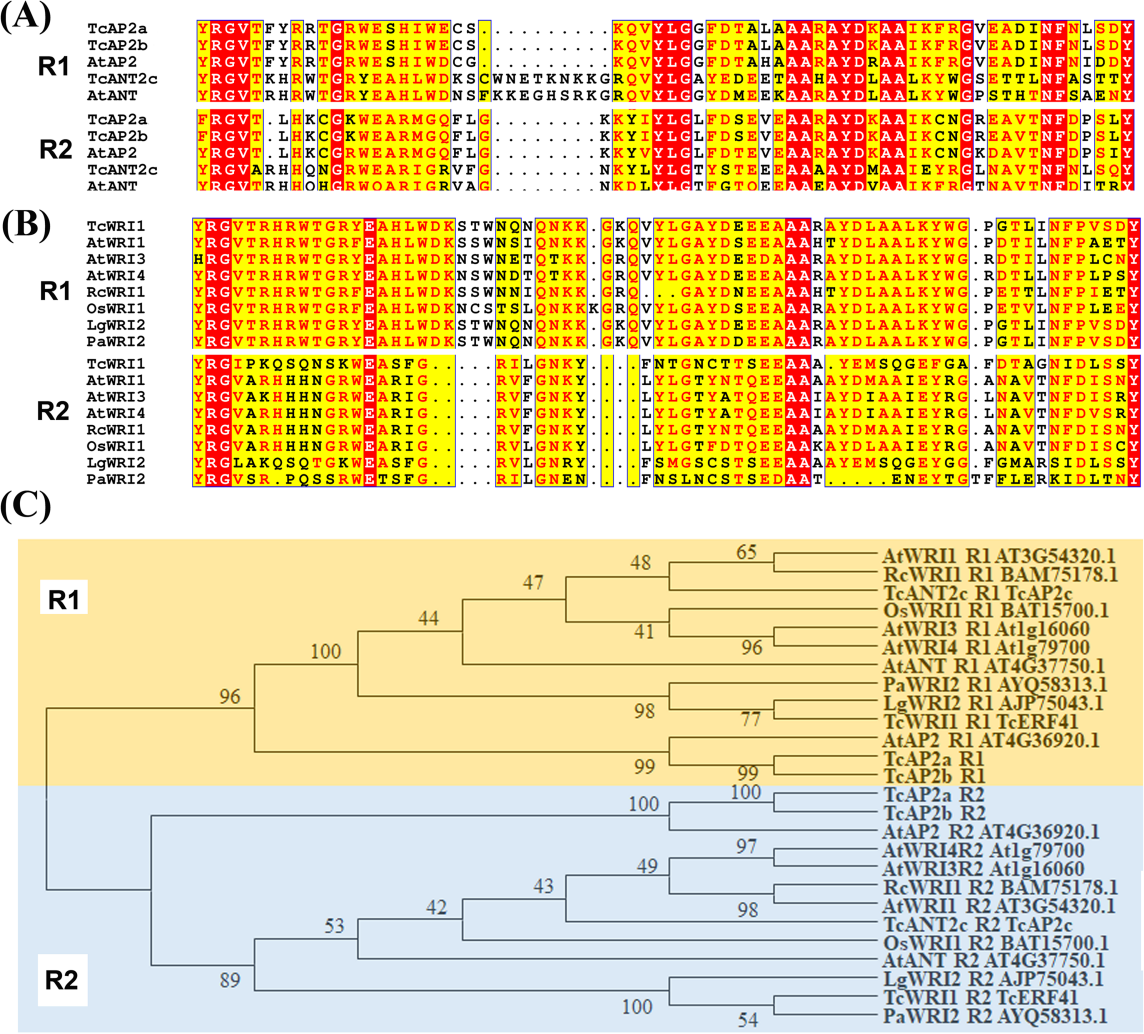
The AP2 domain of AP2 and ANT subgroup members. **a** Alignment of the AP2 domain of AP2s and ANTs, R1 represents the N-terminal AP2 domain while R2 is the C-terminal one. **b** Alignment of the AP2 domain of WRI homologues. The R2 of WRI homologues highly varied their RAYD-motif. **c** Phylogenic tree of the AP2 domain of AP2s and ANTs. R1 and R2 were clustered seperately. The Genbank accession number and TAIR gene ID were listed

Three unigenes named as TcAP2a-c could encode unique proteins with two AP2 domains (Fig. 1a). The R1 of TcAP2c is similar to that of ANT subgroups called TcANT2c (Fig. 1a) [9]. In addition, *TcRAV1* encodes a RAV protein with an extra B3 domain, and 76 proteins contain only one AP2 domain. TcERF41, also named as TcWRI1, is highly conserved with WRI1 proteins and contains only one AP2 domain but has a divergent C-terminal AP2 domain-like motif, which lacks (Y/H/W)LG- and RAYD-motif (Fig. 1b) [32]. AtWRI1/3/4 has two conserved AP2 domains, but the homologues have a highly varied C-terminal AP2 domain (R2) in *Larix gmelinii var. olgensis, Larix kaempferi,* and *Persea Americana* (Fig. 1b) [29]. However, the R2 of WRI homologues is closely related to that of ANTs, indicating that WRI homologues have special evolutionary processes in plants [33] (Fig. 1c).

### New AP2/EREBP with three AP2 domains found in several gymnosperms and bryophytes

**Fig. 2:**
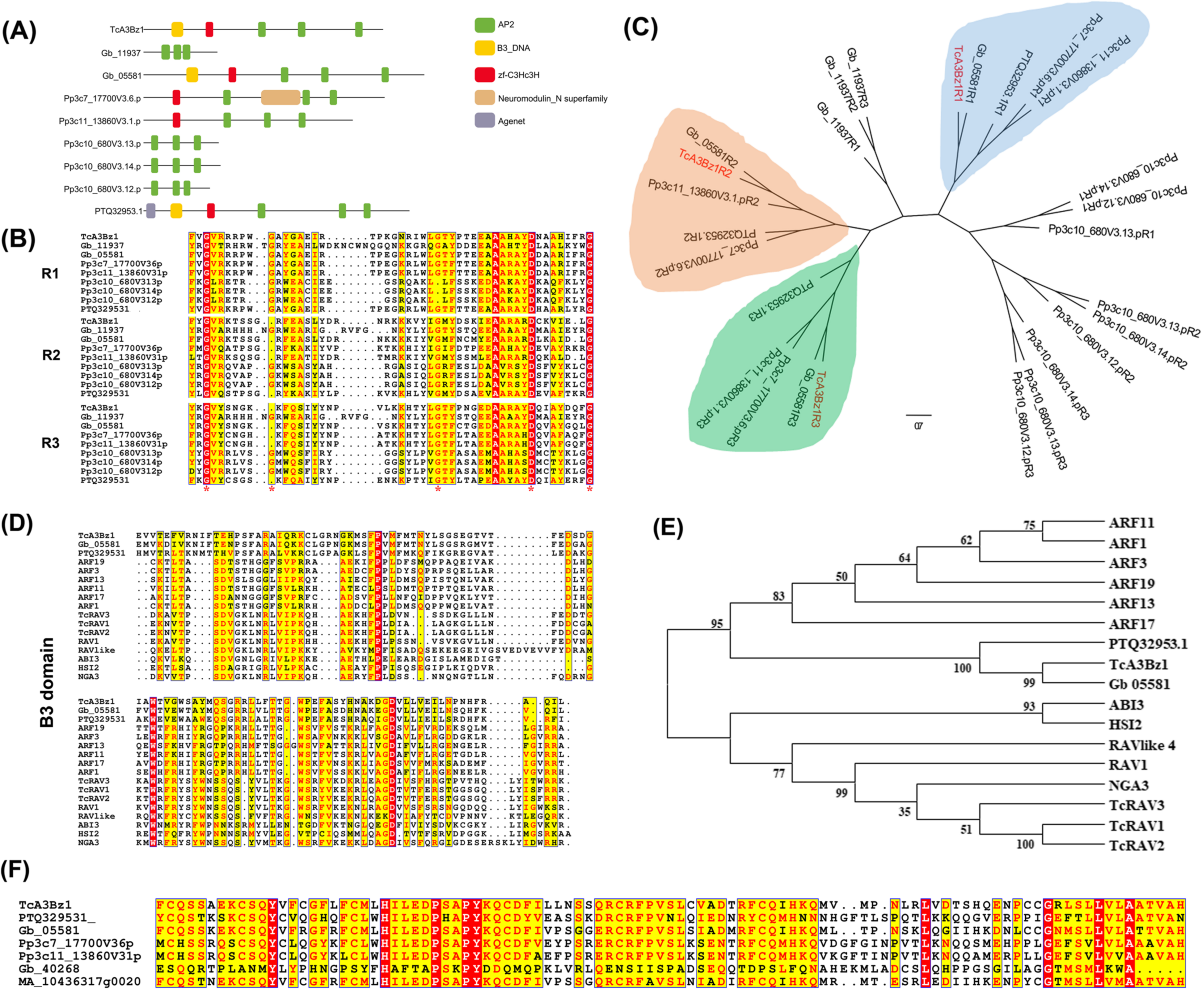
Molecular structure and phylogenic tree of new subgroup members of AP2/EREBPs. TcA3Bz1 and its homologues belong to a new subgroup AP2/EREBPs that contained three AP2-domains. Their molecular structures were highly different. **a** Besides of AP2 domain, each homologues of TcA3Bz1 had different conserved domains, including B3_DNA-binding domain, zf-C3Hc3H-domain, Neuromodullin_N superfamily domain and Agenet domain. **b** Alignment of the three AP2 domains of the new AP2/EREBP subgroup proteins. Red stars indicated the conserved residues of AP2 domain. **c** Phylogenetic analysis of the three AP2 domains. Each repeat of the three AP2 domains of TcA3Bz1, Gb_05581, Pp3c11_13860V3.1.p, Pp3c7_17700V3.6.p and PTQ32953.1 was clustered separately, indicating R1, R2 and R3 separately evolved. **d** Alignment of B3-domains of new clade proteins with several *Arabidopsis thaliana* B3 superfamily proteins. All *Arabidopsis thaliana* B3 superfamily proteins were obtained from TAIR (https://www.arabidopsis.org/browse/genefamily/B3binding.jsp). **e** Phylogenic analysis of B3-domains. **f** Alignment of zf-C3Hc3h-domains of new clade proteins with a MA_10436317g0020 of *Picea abies*. MA_10436317g0020 were highly homologues with N-terminus of TcA3Bz1, without exception that MA_10436317g0020 only contain one AP2-domain

*T. chinensis* has a unigene designated as TcA3Bz1 that encodes a protein containing three AP2, one B3, and one zf-C3Hc3H domains (pfam13891). Different from the divergent C-terminal repeat of AP2 in WRI1 proteins, the three repeats in TcA3Bz1 are all conserved with the common AP2 domain of other proteins (Fig. 2b). Gb_05581 contains the same domains in *G. biloba* (Fig. 2a). In addition, *Gb_11937* also encodes a protein containing three AP2 domains but without B3 or zf-C3Hc3H domain. In *P. patens*, five AP2-EREBP proteins contain three AP2 domains, and two of them contain a zf-C3Hc3H domain each but not the B3. Pp3c7_17700V3.6.p contains a Neurododullin_N superfamily domain between R1 and R2 (Fig. 2a). In gymnosperms *P. abies*, *P. tadae*, *G. montanum*, and pteridophyte *S. moellendorffii*, no protein contains more than two AP2 domains.

After the homologue search of TcA3Bz1 within the NR database (version 2019/6/30), a hypothetical protein MARPO_0093s0032 (GenBank: PTQ32953.1) appears to be the most similar one in *Marchantia polymorpha* and contains three AP2, one B3, and one zf-C3Hc3H domains. Moreover, PTQ32953.1 has an agent domain at the N-terminus of B3 domain (Fig. 2a). After alignment, all the three repeats of AP2 domains seem highly variable. Phylogenic analysis revealed that the R1, R2, and R3 have evolved separately, except for Gb11937 and Pc3Pv3.12-14 (Fig. 2c). The nucleotide sequences of the R2 and R3 repeat of Gb11937 are completely identical, indicating a duplication that leads to three AP2 domains containing a protein (Fig. 2b, Additional file 2).

B3 and zf-C3Hc3H domains are highly conserved in these new clade proteins but not homologous to any reported proteins according to the search within NR database (version 2019/6/30) (E-value >1e-5) (Fig. 2d and f). B3 domains can also be found in RAV proteins but are different from those of the new clade proteins (Fig. 2d). B3-containing proteins are divided into four classes, LAV (Leafy cotyledon2 [LEC2]-abscisic acid insensitive3 [ABI3]–VAL), RAV, ARF (Auxin response factor), and REM (Reproductive meristem), leading to four divergent B3 domains [30, 34]. Among these proteins, the B3 of REM class proteins are distant from the three other classes. The B3 of new clade proteins with three AP2 domains shares low similarities with these four B3 repeats (Fig. 2d). However, phylogenetic analysis suggested that the B3 of new clade proteins and ARFs have a common ancestor though they share low similarities, suggesting that TcA3Bz1 and its homologues could bind similar cis-elements with ARF (Fig. 2e).

All these results showed that AP2/EREBP transcription factors have complicated evolutionary processes. To the best of our knowledge, this study first found the AP2/EREBP proteins with three or more AP2 domains and extra domains such as the zf-C3Hc3H domain, which was first identified in AP2/EREBP proteins. These findings could be interesting study points on the functions of AP2/EREBP transcription factors.

### AP2 domains of DREB and ERF proteins have evolved conservatively in *T. chinensis*

The EREBP subfamily proteins that contain only one AP2 domain in *A. thaliana* could be divided into 13 clades, namely, two subgroups, DREBs (A1–A6), ERFs (B1–B6), and soloists [7]. Many plants only have 1–2 soloists [11, 35–37]. According to our results, no soloist ERFs, which have a conserved HLG-motif in AP2 domain, exist in *T. chinensis* and *P. abies*. However, this protein is highly conserved in *G. biloba*. Hence, the evolutionary process of soloist ERFs in gymnosperms should be further clarified.

**Fig. 3:**
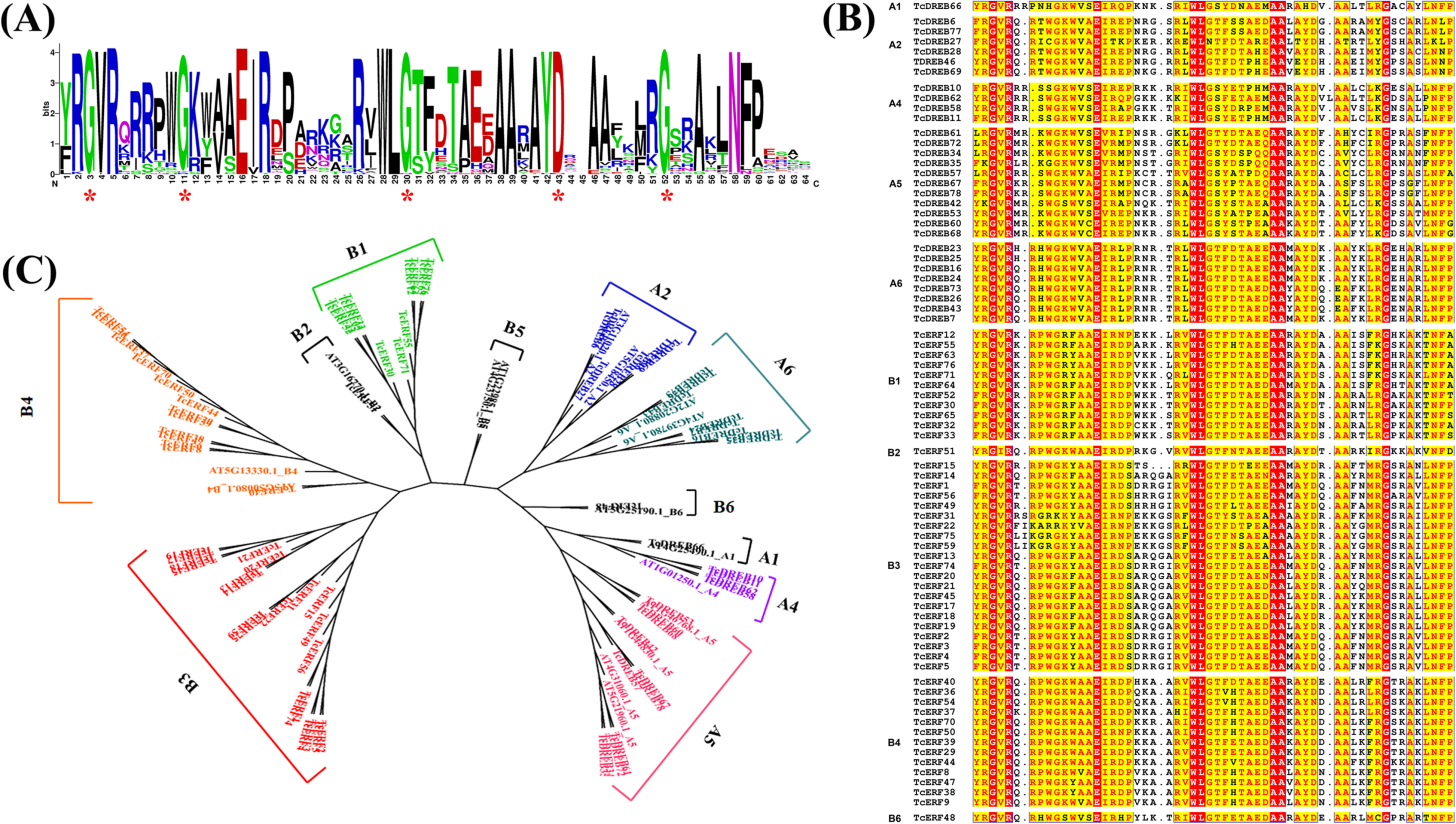
Characteristics of the AP2 domain of *Taxus* DREB and ERF proteins. DREBs and ERFs in *Taxus chinensis* were highly similar with their homolouges in other plants. **a** Logo of the AP2 domain of *Taxus* DREBs and ERFs. **b** Alignment of the AP2 domain of *Taxus* DREBs and ERFs. **c** Phylogenetic analysis of *Taxus* DREBs and ERFs with 16 selected proteins in *Arabidopsis thania*. No B5 and A3 clade proteins were found in *T. chinensis*

The AP2 domain of 76 full-length EREBP proteins of *T. chinensis* was phylogenetically analyzed with 16 representative homologues in *A. thaliana* (Fig. 3c). According to our results, the EREBP subfamily proteins of *T. chinensis* could be divided into 10 clades, and no A3 and B5 members exist in *T. chinensis* [30, 38]. Only one protein belongs to B2, B6, and A1 clades. The B3 clade in *T. chinensis* has 21 ERFs, whereas B4, B1, and A5 groups contain 13, 11, and 10 ERFs, respectively (Fig. 3b). In many plants, B3 is the dominant clade with most members in the ERF subfamily [31, 39–41].

### Conserved motifs in ERF and DREB proteins in *T. chinensis*

Almost all *Taxus* ERFs and DREBs contain the WLG-motif in their AP2 domain, except for TcERF27 that converts it into WLN (Fig. 3b). WLG-motif is 100% identical in all DREBs and ERFs, even in RAVs, but is converted to YLG in AP2 subfamily proteins [31, 42]. The substitution of WLN-motif might infer an interesting biological importance.

**Fig. 4:**
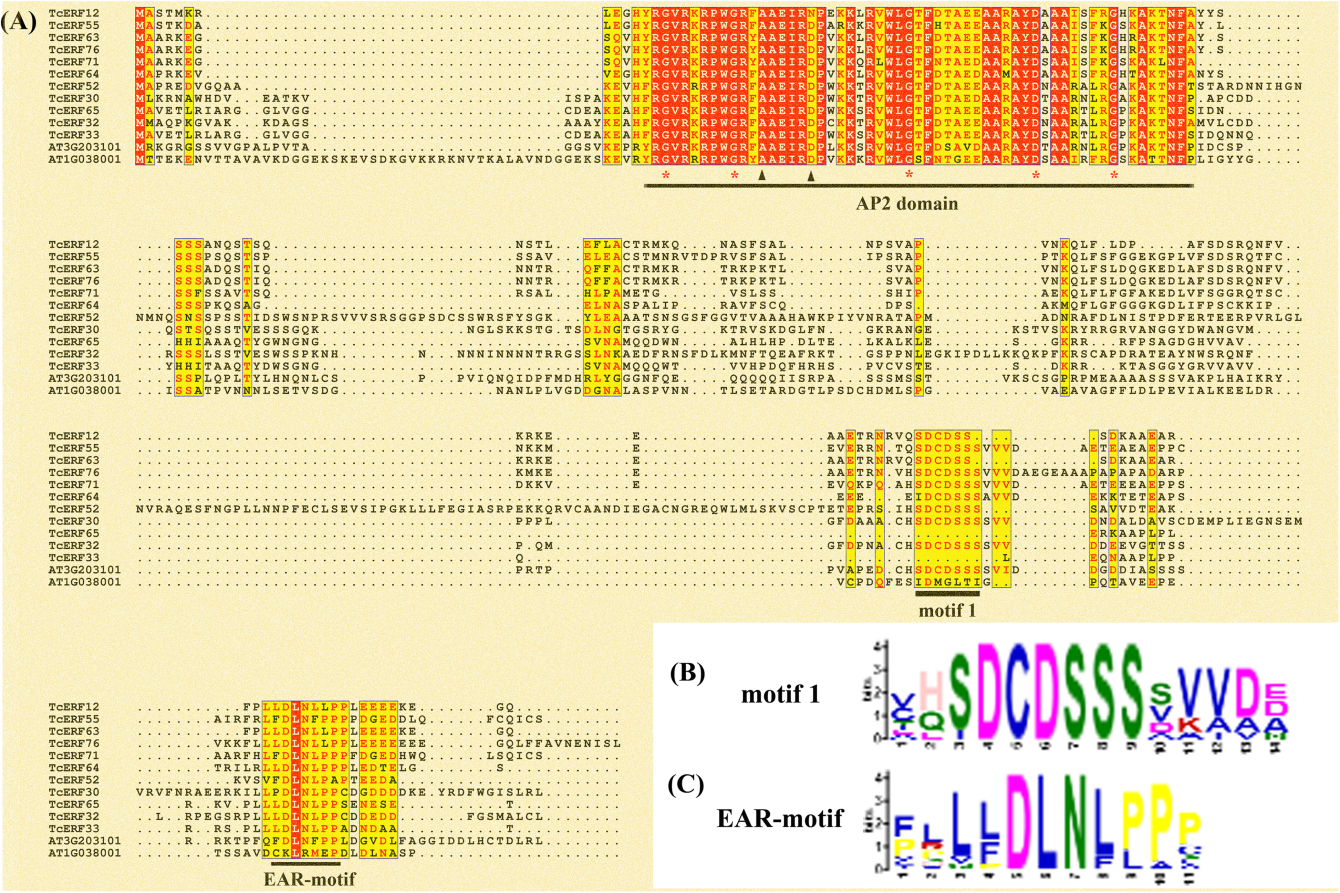
Molecular structure of B1 ERFs. **a** Alignment of B1 ERFs. Besides of the conserved AP2 domain, there were two conserved motifs in B1 ERFs, including motif 1 (**b**) and EAR-motif (**c**)

All B1 TcERF proteins have a (L/F)DLNL/F(X)P-type EAR-motif in their C-terminal, which is commonly present in B1 ERFs [43] and could be considered as an enhanced EAR-motif combination of LxLxLx- and DLNxxP-type that allows physical interaction by TOPLESS co-repressor (Fig. 4a and c) [44–46]. Moreover, a DCDSSS-motif is highly conserved and present at the N-terminal of EAR-motif in most TcERFs, and some AtERFs belong to the B1 clade (Fig. 4a and b).

**Fig. 5:**
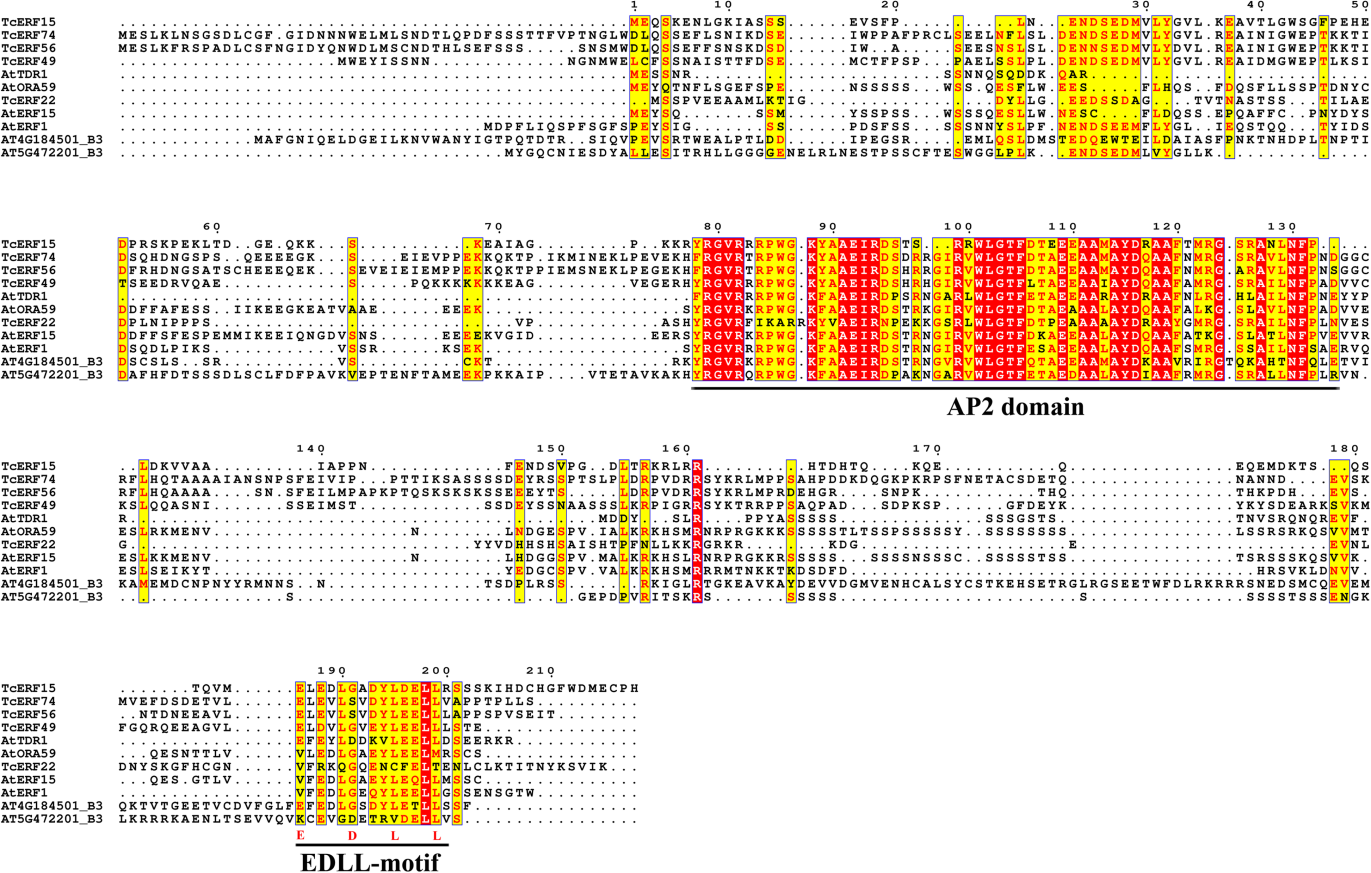
Domain structure of several B3 ERFs that containing EDLL-motif. Sequence alignment of several B3 ERFs that contained EDLL-motif in *Taxus* and *Arabidopsis*. EDLL-motif was not a conserved motif, even the four signature residues were not conserved. Moreover, many B3 ERFs had no EDLL-motif

Several B3 TcERFs have an EDLL motif, which was first found in the C-terminus of AtTDR1 and functions as a strong activation domain [47]. Different from the EDLL motif of unknown B3 ERFs, the D residue is not conserved but substituted by G or S residue in *Taxus* B3 TcERFs. Substitutions are also found in ORA59, ERF1, and ERF15 (Fig. 5). However, the two L residues in this 14 aa motif, which are essential for physical interaction with MED25, are highly conserved (Fig. 5, Additional file 3) [48].

### Expression profiles of AP2/EREBPs in *T. chinensis*

AP2/EREBP superfamily is one of the largest transcription factors in plants and is a crucial regulator of plant development, growth, defense, and series bioactivities [14, 49]. We previously identified two ERFs regulating taxol biosynthesis and obtained a series transcriptomic datasets of *T. chinensis* with varying taxol biosyntheses [15–17]. These datasets were used to elucidate the expression patterns of TcAP2/EREBPs and screen the candidate AP2/EREBP-type regulators of taxol biosynthesis.

**Fig. 6:**
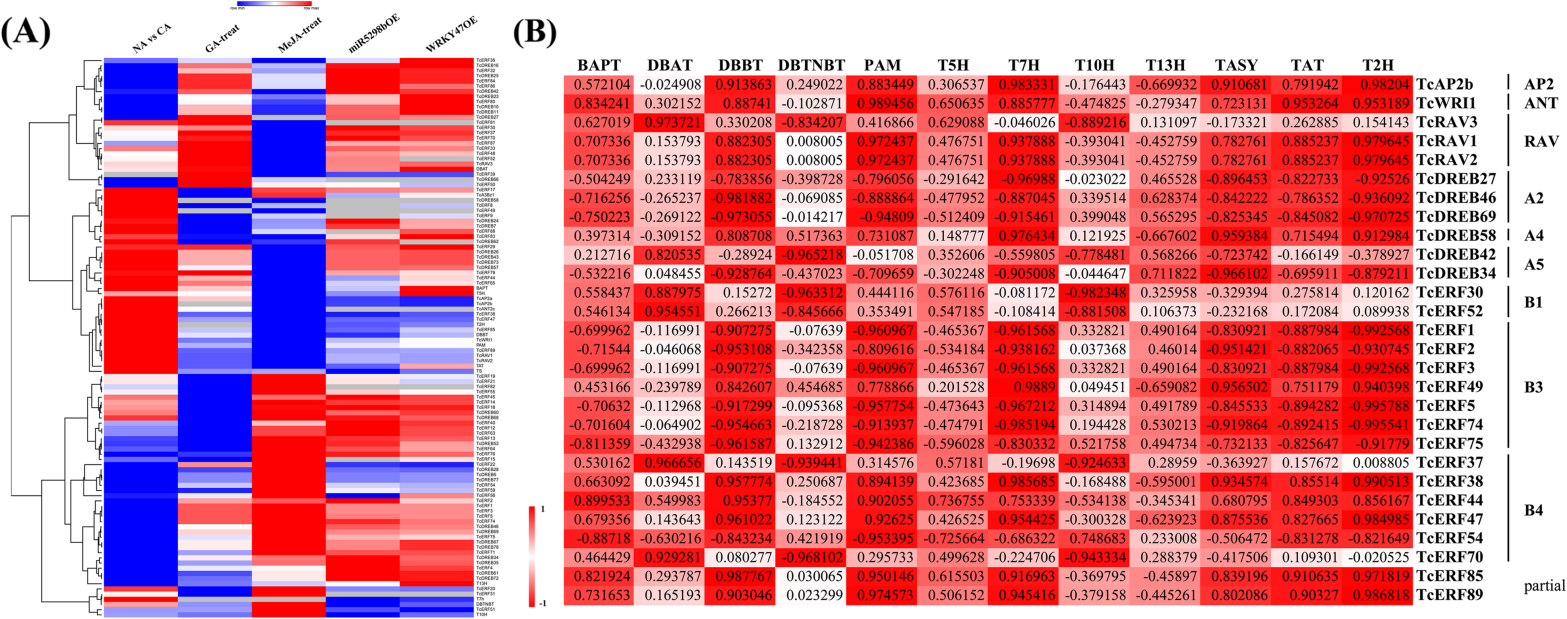
Expression patterns of *Taxus* AP2/EREBP proteins. **a** Expression heatmap of *Taxus* AP2/EREBP proteins. NA means the new induced cells, GA- and MeJA- treated means the cells were treated with related hormones for 24 h and 16 h respectively. And, miR5298bOE and WRKY47OE indicated the samples were transformed into two genes, miR5298 and WRKY47 which were certificated to involve in taxol biosynthesis. **b** Co-expression coefficient values of selected *Taxus* AP2/EREBPs with taxol biosynthesis genes. Only those *Taxus* AP2/EREBPs were chosen which had a coefficient value more than 0.95 with taxol biosynthesis genes. The subgroups of those *Taxus* AP2/EREBPs were indicated, partial means that the proteins were not full-length

Among the 81 full-length AP2/EREBPs, 60, 53, and 66 are downregulated in NA, MeJA-, and GA-treated cells with high production of taxols (Fig. 6a) [50]. However, most B1 members, which function as crucial negative regulators, are downregulated in these datasets, such as 10 (90.9%) members in NA cells, suggesting that B1 plays important roles in these bioactivities and maybe related to the increase in taxol biosynthesis [5]. All three TcAP2s and TcRAV1, might be important in plant development and defense system and are substantially upregulated in NA cells [51]. In addition, 10 out of the 17 differentially expressed B3 members are upregulated in MeJA-treated cells, indicating that B3 TcERFs play vital roles in MeJA response (Fig. 6a, Additional file 2). Moreover, 27 full-length AP2/EREBPs are closely co-expressed with taxol biosynthesis genes with a coefficient value of more than 0.95, indicating that these transcription factors have potential roles in taxol biosynthesis (Fig. 6b).

## Discussion

AP2/EREBP transcription factors, which were named with their conserved AP2 DNA-binding domain, play various roles in developmental processes throughout the entire plant life cycle and are important parts of gene regulatory networks that integrate metabolic, hormonal, and environmental signals in stress acclimation and retrograde signaling in various biological plant activities [4, 5, 49, 51, 52]. AP2/EREBP superfamily proteins were once considered as plant-specific transcription factors. However, several HNH endonucleases were found to contain an AP2 domain, which selectively recognizes the stretches of poly(G)/poly(C) as binding sites in cyanobacterium (*Trichodesmium erythraeum*), ciliate (*Tetrahymena thermophile*), and viruses (*Enterobacteria phage* Rb49 and *Bacteriophage Felix* 01) [53]. In plants, the binding sites of the AP2 domain are also G/C-rich and highly similar to poly(G)/poly(C) stretches [53]. The DNA binding affinity of AP2/EREBP transcription factors indicated the conservative evolution of AP2/EREBP proteins from virus to plants.

In plants, the AP2/EREBP superfamily proteins are commonly divided into AP2, ANT, ERF, DREB, RAV, and soloist subgroups. AP2 and ANT subgroups contain two conserved AP2 domains characterized by a YLG-motif. ERF and DREB have only one WLG-motif containing an AP2 domain. RAV has a B3 domain in addition to the AP2 domain. Soloists have only one AP2 domain with an HLG-motif instead of YLG- and WLG-motifs and are the smallest group in AP2/EREBP proteins, whereas DREB and ERF constitute the largest groups in nearly all plants [8, 11, 30, 35, 39]. These domain structures of AP2/EREBP proteins have evolved conservatively in many plant species, although most of them are angiosperms. However, our results indicated that AP2/EREBP proteins have highly different evolutionary processes and domain structures in gymnosperms and bryophytes.

A new clade of AP2/EREBP proteins wasdiscovered to have three conserved AP2 domains in *T. chinensis*, *G. biloba*, *P. patens*, and *M. polymorpha* (Fig. 2). However, fern *S. moellendorffii* has no such subgroup members. This kind of AP2/EREBP proteins is not found in all gymnosperms, such as *P. abies*, *P. tadae*, and *G. montanum*. Moreover, the proteins of the new subgroup have different domain structures constituting other conserved domains, such as B3 and zf-C3Hc3H domains. After identification via transcriptome datasets, *T. chinensis* has only one protein, named as TcA3Bz1, which contains three AP2, one B3, and one zf-C3Hc3H domains, and this homologue of TcA3Bz1 was only found in *M. polymorpha and G. biloba*. B3 domain was found at the N-terminal of the three AP2 domains, whereas it is always located at the C-terminal in RAV proteins [54]. In *G. biloba* and *P. patens*, most proteins belong to the new clade with only three AP2 domains. Two other proteins of *P. patens* contain an extra zf-C3Hc3H domain at the N-terminus of the three AP2 domains. In summary, the new subgroup of AP2/EREBP proteins only exists in *Taxus* spp., *G. biloba*, and bryophytes, and these proteins have diverse function domains and thus have evolved differently in higher plants.

B3 domain is an essential element for DNA-binding, but different classes bind various cis-elements; for instance, ARF binds to 5′-TGTCTC-3′, and RAV1 recognizes 5′-CACCTG-3′ [30]. Although the B3 of TcA3Bz1 has a common ancestor with ARF, they share few similarities, resulting in their different binding properties [30]. In particular, the RGQP(K/R)R-motif in ARF B3 domain, which is essential for ARF to recognize related cis-elements, is absent in the TcA3Bz1 B3 domain (Fig. 2d), suggesting that the latter domain binds an unknown cis-element [30]. zf-C3Hc3H domains are highly conserved in almost all new clade proteins, but minimal information is available on this domain, except that it is DNA binding [51, 55]. In summary, these new clade proteins, such as TcA3Bz1, might play novel functionalities in plants.

Each subgroup of AP2/EREBP proteins is highly conserved in *T. chinensis* and angiosperms, and only the soloist type is not present. The AP2 domain of each subgroup bind differently with various DNA cis-elements and functions [53]. The YRG- and RAYD-motifs of the AP2 domain are responsible for DNA binding and protein–protein interaction, respectively [52]. The first residues Y and R would sometimes differentiate into F and M/L, respectively, in a few types of proteins [42, 56]. In addition, (H/Y/W)LG-motif is the characteristic conserved motif that distinguishes these AP2/EREBP subgroups, YLG in AP2 and ANT, HLG in soloist, and WLG in DREB, ERF, and RAV [11, 31, 57]. In *T. chinensis*, YRG-, (Y/W/H)LG-, and RAYD-motifs are also highly conserved in common subtype proteins. The TcAP2a&b of AP2 clade and TcAP2c of ANT clade both contain the YLG-motif that is conserved in all AP2 domains of AP2 and ANT proteins [9, 31, 57]. Most TcDREBs, TcERFs, and TcRAVs have the WLG-motif.

One difference is that the first residue of YRG- and RAYD-motif varies between *T. chinensis* and angiosperms, especially in RAYD-motif where R is substituted by L/M/V/K/E/Q/I/Y/H (Fig. 3). Determining the influence of highly variable RAYD-motif in *T. chinensis* is difficult due to the limited information about the molecular basis of the interactions of RAYD-motif and related proteins. (Y/W/H)LG-motif exhibits better conservatism than YRG- and RAYD-motifs in *T. chinensis*, and only two proteins have different motifs, including the WLN- of TcERF27 and NTG-motif of R2 (C-terminal AP2 domain) of TcWRI1. All these results indicate that the (Y/W/H)LG-motif has an important role in AP2 domain functions (Fig. 1) [57].

The 14th and 19th residues differ between DREB (V14, E19) and ERF (A14, D19), though the 19th residue is not highly conserved sometimes [5, 35, 58]. The appearance of TcDREBs and TcERFs is also in accordance with these results, and only TcERF8&9 (B4) and TcERF48 (B6) have a V14 residue similar to DREBs (Fig. 3). Further verification might help clarify the effects of V substitution in TcERF8&9&48.

Despite the differences observed, the AP2 domain evolution is highly conserved in *T. chinensis* and angiosperm plants [6, 11, 30, 31, 35, 36, 38–41]. In conclusion, four G (two Gs from YRG- and WLG-motif, two Gs from G11 and G52, Fig. 3a) and one D residue from RAYD-motif are extremely conserved and thus are essential for AP2 domains (Fig. 3). However, in these new subgroup proteins, the three conserve motifs and the four residues of AP2 domain are all variable (Fig. 2). The existence and diverse domain structure of the new AP2/EREBP factors with three AP2 domains indicated the complexity of the evolutionary processes of AP2/EREBP proteins in angiosperm plants and other higher plants.

The EAR- and EDLL motifs of B1 and B3 ERFs, respectively, are also highly conserved in *T. chinensis*.

With the presence of EAR-motif, B1 ERF functions as negative regulators in plants. In *T. chinensis*, all B1 TcERFs have the (L/F)DLNL/F(X)P-type EAR-motif, which is highly conserved in all B1 ERFs from other plants [43]. The EAR-motif generally has two types, LxLxL- and DLNxxP-types, such as JAZ, AUX/IAA, and NINJA [41, 46]. (L/F)DLNL/F(X)P-type could be considered as an enhanced EAR-motif combination of the two types and could strengthen the negative regulation functions. TcERF12, a B1 ERF transcription factor, has a (L/F)DLNL/F(X)P-type EAR-motif and was previously confirmed as a negative regulator of *TASY* gene in taxol biosynthesis (Fig. 4) [5].

EDLL motif, a short motif characterized by four discrete residues, has a strong activation domain and was first found in AtERF98 [47, 48]. Four B3 TcERFs contain a highly similar EDLL motif with AtTDR1 at their C-terminus, including TcERF15, a positive regulator of taxol biosynthesis (Fig. 5) [5]. Further studies revealed that the two L residues, especially the L228 of ORA59, are essential for the activation by binding with MED25, whereas the E and D residues are not conserved [48, 59]. Although not all B3 ERFs, including TcERFs, have conserved EDLL motif, most of them function as positive regulators; for instance, CrORCA3 does not contain an C-terminus EDLL motif but could activate the expression of *STR* gene (Additional file 3) [4]. The mechanism of these B3 ERFs without an EDLL motif should be further clarified.

Many reports characterized the AP2/EREBP transcription factors in various plants; the AP2 domain for DNA-binding is highly conservative across plant kingdoms [11, 39, 41]. Moreover, nearly all subtype proteins of AP2/EREBP factors, including AP2, ANT, RAV, DREB and ERF, are present from *Physcomitrella patens* to angiosperms. The high number of classifications concluded the same evolutionary process of AP2/EREBP transcription factors, but the identification of new clade proteins reveals a different evolution process of AP2/EREBPs in higher plants. This finding might be an important guidance for us to re-recognize the speciation in plant kingdoms.

In addition to the three conserved AP2 domains, two DNA-binding domains, a new B3 domain and a new zf-C3Hc3H domain, showed extreme conservation in all new subtype AP2/EREBP proteins and were first identified in this study. Additional DNA-binding domains may provide novel functionalities for these new clade proteins by regulating downstream genes. However, identifying the roles of the new clade proteins in plants is difficult due to the uncharacterized B3- and zf-C3Hc3H domains, which were highly different form known related domains. Given its absence in angiosperms, the new clade AP2/EREBPs may play specific roles in *Marchantia polymorpha* and several gymnosperms. Such mutant lines and genetic manipulations of *Marchantia polymorpha* and several gymnosperms are required to clarify the functions of these AP2/EREBPs with newly domain structures.

## Conclusions

Identification and evolutionary analysis revealed that AP2/EREBP transcription factors have complex evolutionary processes during plant divergence. The newly found proteins, which have three conserved AP2 domains, exhibited irregularity among higher plants. To our knowledge, no such report was found for angiosperms and pteridophyte *S. moellendorffii*. Not all gymnosperm plants have new AP2 clade proteins; *T. chinensis* and *G. biloba* contain this protein, whereas *Picea abies*, *Pinus tadae* and *G. montanum* do not. For bryophytes, *P. patens* and *M. polymorpha* have these new proteins. Moreover, the domain structure of these new AP2 proteins varies in these plants. These results indicate that the proteins of this new AP2 clade must have a special evolutionary process in plants, but the other clades of AP2/EREBPs have evolved conservatively in *T. chinensis* and other plants. The new subgroup proteins might have separately evolved from the common groups, and angiosperms and several gymnosperms might have abandoned the new-type AP2/EREBPs while diverging.

## Supplementary information

**Additional file 1. **Protein sequences of *Taxus* AP2/EREBPs.

**Additional file 2. **Sequence alignment of all B3 ERFs. *Arabidopsis* B3 ERF were obtained from TAIR (https://www.arabidopsis.org/), while CrORCA3 downloaded from UniprotKB under accession number Q9LDB6. This CrORCA3, lacked of the EDLL-motif, was certificated to up-regulate biosynthesis of terpenoid indole alkaloids in *Catharanthus roseus*.

**Additional file 3.** Partial nucleotide sequence of Gb_11937. The nucleotide sequence of Gb_11937 encoded R2 and R3 of AP2 domain were completely identical. The red characters encoded the R2 and R3 repeat of AP2 domain, the yellow ones were also completely identical, indicating a duplication.

**Additional file 4. **Nucleotide sequences of *Taxus* AP2/EREBPs.

## Data Availability

Plant materials, *Taxus chinensis* (Pilger) Rehd*.* (deposited the voucher specimen in Herbarium of Wuhan Botanical Garden of CAS under accession number HIB0087305 and identified by Shuxia Fu), obtained from the Huawei Seedling Farmers’ Professional Cooperative (Xianning, China), who provided permission to use the seedlings for our scientific research. The *Taxus* cells were cultivated in our lab. Raw sequencing data were submitted in GEO database (accession numbers: GSE28539) and SRA databases (accession numbers: SRR1339463, SRR1339474, SRR1343578, and SRR8083193 to SRR8083198). All the proteins in *Taxus chinensis* we used could obtained in Additional file 1. The others were get from public databases, PTQ32953.1 (accession number) from GenBank (https://www.ncbi.nlm.nih.gov/protein/PTQ32953.1/), the genomes of *Physcomitrella patens*, *Selaginella moellendorffii* were from JGI (https://phytozome.jgi.doe.gov/pz/portal.html#). *Arabidopsis* AP2/EREBP proteins were searched from plnTFdb database (http://plntfdb.bio.uni-potsdam.de/v3.0/), *Picea abies* and *Pinus tadae* were obtained from ConGenIE (http://congenie.org/), *Ginkgo biloba* was from Giga (http://gigadb.org/dataset/100209) and *G. montanum* was downloaded from DRYAD (https://datadryad.org/resource/doi:10.5061/dryad.0vm37.2).

## Abbreviations

ANT: AINTEGUMENTA
AP2: APETELA2
DREB: Dehydration responsive element binding protein
EREBP: Ethylene-responsive element binding protein
ERF: Ethylene-responsive factor
GA: Gibberellin acid
HMM: Hidden Markov Model
MeJA: Methyl jasmonate
RAV: related to ABI3/VP1
TASY: Taxadiene synthase
