# Supplementary material for: New different origins and evolutionary processes of AP2/EREBP transcription factors in Taxus chinensis
Source: BMC Plant Biol. 2019 Oct 7;19:413. doi: 10.1186/s12870-019-2044-z (PMC6781369; doi:10.1186/s12870-019-2044-z)
Supplement: Supplementary file 2 — Additional file 2. Sequence alignment of all B3 ERFs. Arabidopsis B3 ERF were obtained from TAIR (https://www.arabidopsis.org/), while CrORCA3 downloaded from UniprotKB under accession number Q9LDB6. This CrORCA3, lacked of the EDLL-motif, was certificated to up-regulate biosynthesis of terpenoid indole alkaloids in Catharanthus roseus. [file 12870_2019_2044_MOESM2_ESM.pdf]

```
                670       680       690       700       710       720
661     GTCTCGAAGGAAGAATACCTGGCTTCTCTCAGGAGAAGAAGCAGCGGTTTCTCAAGAGGA
221      V  S  K  E  E  Y  L  A  S  L  R  R  R  S  S  G  F  S  R  G

                730       740       750       760       770       780
721     GTTTCAAAGTACCGCGGTGTGGCAAGACATCATCACAATGGACGCTGGGAAGCACGAATT
241      V  S  K  Y  R  G  V  A  R  H  H  H  N  G  R  W  E  A  R  I

                790       800       810       820       830       840
781     GGCCGTGTGTTTGGCAATAAGTATCTCTACTTGGGAACCTACAGCACCCAAGAAGAAGCA
261      G  R  V  F  G  N  K  Y  L  Y  L  G  T  Y  S  T  Q  E  E  A

                850       860       870       880       890       900
841     GCCGCAGCCTACGACATGGCCGCCATAGAATACAGAGGCTTAAACGCTGTGACAAACTTT
281      A  A  A  Y  D  M  A  A  I  E  Y  R  G  L  N  A  V  T  N  F

                910       920       930       940       950       960
901     GATTTGAGTCGCTACATAGGAAGAAGCAGCGGTTTCTCAAGAGGAGTTTCAAAGTACCGC
301      D  L  S  R  Y  I  G  R  S  S  G  F  S  R  G  V  S  K  Y  R

                970       980       990      1000      1010      1020
961     GGTGTGGCAAGACATCATCACAATGGACGCTGGGAAGCACGAATTGGCCGTGTGTTTGGC
321      G  V  A  R  H  H  H  N  G  R  W  E  A  R  I  G  R  V  F  G

               1030      1040      1050      1060      1070      1080
1021    AATAAGTATCTCTACTTGGGAACCTACAGCACCCAAGAAGAAGCAGCCGCAGCCTACGAC
341      N  K  Y  L  Y  L  G  T  Y  S  T  Q  E  E  A  A  A  A  Y  D

               1090      1100      1110      1120      1130      1140
1081    ATGGCCGCCATAGAATACAGAGGCTTAAACGCTGTGACAAACTTTGATTTGAGTCGCTAC
361       M  A  A  I  E  Y  R  G  L  N  A  V  T  N  F  D  L  S  R  Y  I

               1150      1160      1170      1180      1190      1200
1141    ATAGGGTGGCTAAGACCCGGCCAAAACACTAGTTCAAGTGATCAGGAATCAGGATCGAGT
381       G  W  L  R  P  G  Q  N  T  S  S  S  D  Q  E  S  G  S  S
```
