# Supplementary material for: New different origins and evolutionary processes of AP2/EREBP transcription factors in Taxus chinensis
Source: BMC Plant Biol. 2019 Oct 7;19:413. doi: 10.1186/s12870-019-2044-z (PMC6781369; doi:10.1186/s12870-019-2044-z)
Supplement: Supplementary file 3 — Additional file 3. Partial nucleotide sequence of Gb_11937. The nucleotide sequence of Gb_11937 encoded R2 and R3 of AP2 domain were completely identical. The red characters encoded the R2 and R3 repeat of AP2 domain, the yellow ones were also completely identical, indicating a duplication. [file 12870_2019_2044_MOESM3_ESM.pdf]

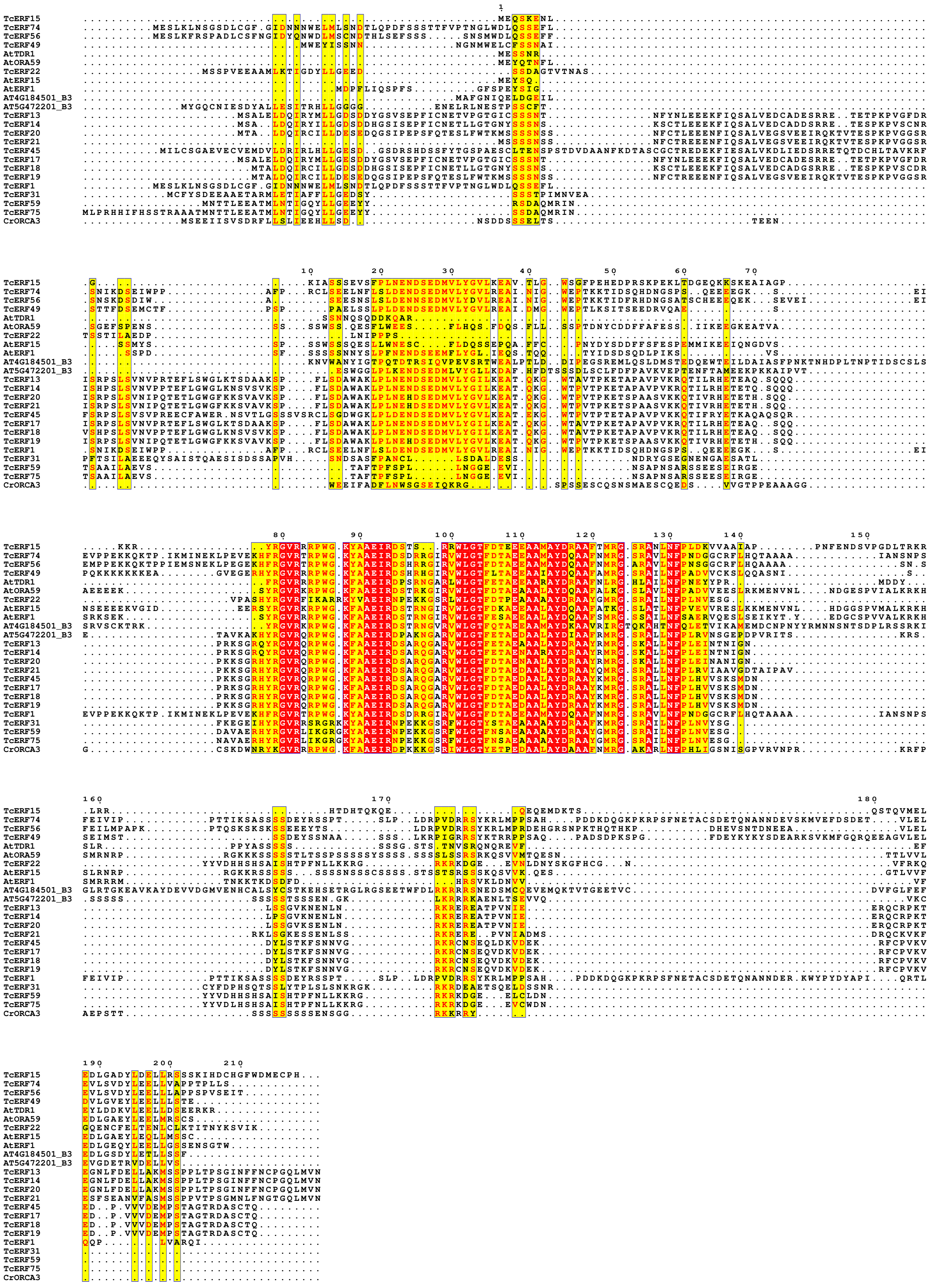
TcERF15
TcERF74
TcERF56
TcERF49
AtTDR1
AtORA59
TcERF22
AtERF15
AtERF1
AT4G184501_B3
AT5G472201_B3
TcERF13
TcERF14
TcERF20
TcERF21
TcERF45
TcERF17
TcERF18
TcERF19
TcERF1
TcERF31
TcERF59
TcERF75
CrORCA3
1
10
20
30
40
50
60
70
80
90
100
110
120
130
140
150
160
170
180
190
200
210
